# Can hunting data be used to estimate unbiased population parameters? A case study on brown bears

**DOI:** 10.1098/rsbl.2016.0197

**Published:** 2016-06

**Authors:** Martin Leclerc, Joanie Van de Walle, Andreas Zedrosser, Jon E. Swenson, Fanie Pelletier

**Affiliations:** 1 Département de biologie & Centre for Northern Studies, Université de Sherbrooke, Sherbrooke, Quebec, Canada; 2 Department of Environmental and Health Studies, University College of Southeast Norway, Bø, Norway; 3 Institute of Wildlife Biology and Game Management, University of Natural Resources and Life Sciences, Vienna, Austria; 4 Department of Ecology and Natural Resource Management, Norwegian University of Life Sciences, Ås, Norway; 5 Norwegian Institute for Nature Research, Trondheim, Norway

**Keywords:** harvest, hunting regulation, temporal trends, Sweden, *Ursus arctos*

## Abstract

Quantifying temporal changes in harvested populations is critical for applied and fundamental research. Unbiased data are required to detect true changes in phenotypic distribution or population size. Because of the difficulty of collecting detailed individual data from wild populations, data from hunting records are often used. Hunting records, however, may not represent a random sample of a population. We aimed to detect and quantify potential bias in hunting records. We compared data from a long-term monitoring project with hunting records of brown bears (*Ursus arctos*) in Sweden and investigated temporal trends (1996–2013) in the ratio of yearlings to adult females, yearling mass and adult female mass. Data from hunting records underestimated the decline in yearling and adult female mass over time, most likely owing to the legal protection of family groups from hunting, but reflected changes in the ratio of yearlings to adult females more reliably. Although hunting data can be reliable to approximate population abundance in some circumstances, hunting data can represent a biased sample of a population and should be used with caution in management and conservation decisions.

## 1. Introduction

Unbiased sampling is required to detect changes in population size or age and sex structure. For example, information on individually marked animals can be used to estimate population trends. Such data, however, are not always available, owing to the high costs and logistic difficulties of monitoring programmes. Therefore, large datasets from hunting records are commonly used to obtain biological information [1,2]. This practice has been criticized, because data from hunt-killed animals may be biased [3], because hunters almost always select individuals from populations non-randomly, selecting primarily adults, sometimes as large as possible. For example, Martínez *et al*. [4] showed that different hunting strategies select for different body masses in a red deer (*Cervus elaphus*) population. Pelletier *et al*. [5] showed that data from trophy-hunted bighorn sheep (*Ovis canadensis*) underestimated temporal declines in horn length. Festa-Bianchet *et al*. [6] showed with simulations that trophy record books underestimate increasing trends in horn length and do not detect declines in horn length. These biases, however, have mostly been reported for morphological traits targeted through trophy hunting (legal definition or preferences of hunters to shoot an animal based on a morphological trait) and may not exist under less selective hunting regimes [7].

Assessing the accuracy of hunting record data to estimate population parameters is difficult, as it requires a hunted population that is also the subject of intensive unbiased, longitudinal monitoring research, which is seldom the case [8]. To the best of our knowledge, only two populations fulfil these requirements: bighorn sheep in Canada (trophy hunting) and brown bears (*Ursus arctos*) in Scandinavia. We used data from the individual-based, long-term monitoring by the Scandinavian Brown Bear Project (SBBP), which captured and marked 50–80% of the bear population, and data from hunting records in the same area in Sweden. We aimed to compare temporal trends from both datasets to explore and quantify biases in hunting records. We focused our analyses on proxies of recruitment and individual condition commonly used by managers to assess population performance [9]: ratio of yearlings to adult females, yearling mass and adult female mass. Trophy hunting for bears is rare in Sweden, and hunting mortality rates are similar between sex- and age-classes [10,11]. However, family groups (females with dependent offspring) are legally protected from hunting [11] and might cause a non-random sampling of the killed individuals, as heavier females might reproduce more than lighter females. Therefore, hunting records might be biased, causing differences in temporal trends between the monitored bears and hunting records.

## 2. Methods

We used data collected by the SBBP in southcentral Sweden (Dalarna and Gävleborg counties). The main method of the SBBP is to capture, mark and weigh mothers and their yearling offspring after den emergence in spring, and to follow these individuals as long as possible, preferably for life (50–80% of females are marked in the study area). Marked adult females are recaptured and weighed every 2–3 years, depending on their reproductive status. Young bears can be hunted after weaning in June–July (either as yearlings (79% of litters) or as 2 year olds [12]). See electronic supplementary material, appendix S1 for further details on capture and monitoring.

Bears are hunted during autumn in Sweden. The hunting season ends when the quota is reached, but there is no limit on the number of bears that an individual hunter can kill [11]. Hunters are required to report all bear carcasses for a compulsory inspection on the day of kill to record the bear's sex and body mass [11]. A premolar is extracted for age determination [11]. We used data from bears shot in Dalarna and Gävleborg counties from 1996 to 2013 to spatio-temporally match data from monitored bears (see electronic supplementary material, appendix S2).

We calculated annual ratios of yearlings to adult females (greater than or equal to 4 years old [13]) and used generalized linear models with binomial error distribution to assess differences in the temporal trend between hunting and monitoring datasets. We used linear models to test the temporal trend for yearling mass and included sex as a covariate to account for sexual dimorphism [14]. Yearling mass was log-transformed to fulfil statistical assumptions. We scaled (mean = 0, variance = 1) the mass of monitored and hunter-killed bears separately to account for the fact that these measures were taken in spring and autumn, respectively. The initial models included ‘year’ and the interaction between ‘year’ and bear ‘status' (monitored or hunter-killed) to test for different temporal trends between datasets (see table 1 for model descriptions).

**Table 1.** Final models obtained by backward selection to compare hunting records and monitoring data of brown bears in Sweden, 1996–2013. Response variables are: ratio of yearlings to adult females (*a*), scaled log(yearling mass) (*b*) and scaled adult female mass (*c,d*).

variable	coefficient	s.e.	statistic	*p*-values
(*a*) ratio of yearlings (McFadden *R*^2^ = 14.8%)			*z*-value	
intercept	96.069	32.353	2.97	0.003
year	−0.048	0.016	−2.96	0.003
status hunter-killed	−0.493	0.163	−3.02	0.003
variables removed: year × status (*p*-value = 0.31)				
(*b*) yearling mass (*R*^2^ = 21.4%)			*t*-value	
intercept	221.5	23.30	9.50	<0.001
sex male	0.312	0.092	3.40	0.001
status hunter-killed	−132.6	43.60	−3.04	0.003
year	−0.111	0.012	−9.51	<0.001
year × status hunter-killed	0.066	0.022	3.05	0.002
variables removed: none				
(*c*) hunter-killed adult female mass (*R*^2^ = 11.1%)			*t*-value	
intercept	−0.749	0.175	−4.29	<0.001
age	0.091	0.019	4.75	<0.001
variables removed: age^2^ (*p*-value = 0.77) and year (*p-*value = 0.21)				
(*d*) Monitored adult female mass (*R*^2^ = 50.0%)			*t*-value	
intercept	158	23	6.89	<0.001
age	0.244	0.045	5.38	<0.001
age^2^	−0.006	0.002	−2.89	0.004
year	−0.079	0.011	−6.95	<0.001
variables removed: none				

To evaluate temporal trends in adult female mass, the analyses of hunter-killed and monitored bears were performed separately, because monitored females were measured repeatedly, unlike hunter-killed females. Adult female mass in both datasets was scaled to facilitate comparison of model slopes. We used linear models to evaluate trends in the mass of hunter-killed adult females and linear-mixed models (random intercept: female identity) for monitored females. The initial models (table 1) included ‘year’, ‘age’ and ‘age^2^’ to test for nonlinear effects of age. All statistical analyses were performed using backward selection to remove non-significant effects [15] with R 3.2.2 [16].

## 3. Results

**Figure 1.**
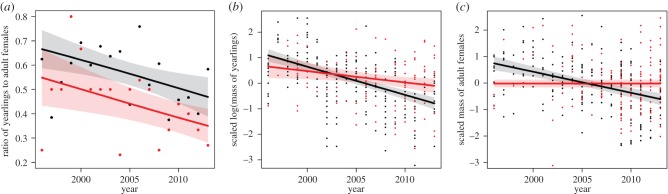
Predictions (solid line) and 95% confidence intervals from the final models comparing data from hunting records (red line) with monitored bears (black line) for the ratio of yearlings to adult females (*a*), scaled log(yearling mass) ((*b*); prediction for male), and scaled adult female mass ((*c*); prediction for 8 year old female) in Sweden, 1996–2013. See Methods for information on scaling procedure.

Hunting records included 108 yearlings and 157 adult females, and the monitoring data included 266 yearlings and 82 adult females weighed between one and six times, for a total of 205 body masses (electronic supplementary material, appendix S3). We found a decline in the ratio of yearlings to adult females over time in both the monitoring dataset and the hunting records (table 1*a* and figure 1*a*). However, this ratio was significantly lower in the hunting records (table 1*a*). Body mass of monitored and hunter-killed yearlings decreased significantly over time (table 1*b* and figure 1*b*), but the mass of hunter-killed yearlings declined at a significantly faster rate than the mass of monitored yearlings (table 1*b*). From 1996 to 2013, the mean mass of monitored and hunter-killed yearlings decreased by 43% (12.5 kg) and 17% (10.2 kg), respectively (electronic supplementary material, appendix S4). The mass of hunter-killed adult females showed no temporal trend, but that of adult female monitored bears declined significantly over time (table 1*c,d* and figure 1*c*). From 1996 to 2013, the mean monitored mass of adult females decreased by 23% (22.6 kg; electronic supplementary material, appendix S5).

## 4. Discussion

We showed that hunting data can differ significantly from monitoring data. Although temporal trends in bear hunting records and monitored data were similar in direction in two-thirds of the cases and proved reliable when evaluating a decline in the ratio of yearlings to adult females, they underestimated the decline in yearling and adult female mass observed in the monitoring data.

Although datasets were recorded at different times of the year (but see electronic supplementary material, appendix S6), differences between the datasets likely reflected bias caused by hunting regulations and ongoing ecological changes. Bear population density has increased during the study period [17]. Density-dependent factors (e.g. food availability [18]) might explain the decline of mass of yearlings and adult females in the monitoring dataset (figure 1). The temporal trends observed in the hunting data, however, did not always match the pattern observed in the monitoring data. In Sweden, all bears can be shot legally, except family groups [11], which may have skewed the hunting data. As yearlings of low mass are more likely to stay with their mother for a second year [12], an under-representation of yearlings of low mass would be expected in the hunting data. Similarly, small adult females might have a lower probability of weaning their offspring as yearlings [12,14]. Therefore, small females might be less available to hunters. Hunting data showed a consistent bias over time in the ratio of yearlings to adult females, which could be explained by an approximately 10% yearling mortality that occurs during summer [10]. In recent years, weaning age has increased, with fewer offspring weaned as yearlings and a higher proportion weaned as 2.5 year olds (SBBP 2016, unpublished data). This leads to a reduction in the ratio of yearlings in both datasets. However, as both the offspring and mother in a family group are protected from hunting, longer maternal care also implies a lower number of adult females available to hunt, which should prevent further bias in the yearling/female ratio.

Obtaining accurate information on population parameters is critical to establish management plans that ensure sustainable exploitation of wild species. Depending on the hunting system and population parameter studied, the use of hunting records can sometimes be reliable [9,19]. However, our results showed that hunting records should be used cautiously when quantifying fluctuations in individual condition and population recruitment. To ensure that observed trends reflect true population processes, bias should be estimated [20] whenever possible. This could be achieved through a parallel longitudinal monitoring of a subsample of the population. If such monitoring is not possible, then simulations based on hunting data could be useful to evaluate if hunting data can detect changes in population trends and parameters [6] and be used in management and conservation decisions.

## Supplementary Material

Supplementary materials

## Supplementary Material

Data

## Data accessibility

The datasets supporting this article have been uploaded as part of the electronic supplementary material.
