# Supplementary material for: Can hunting data be used to estimate unbiased population parameters? A case study on brown bears
Source: Biol Lett. 2016 Jun;12(6):20160197. doi: 10.1098/rsbl.2016.0197 (PMC4938049; doi:10.1098/rsbl.2016.0197)

Can hunting data be used to estimate unbiased population parameters?
A case study on brown bears.

Biology Letters

Martin Leclerc, Joanie Van de Walle, Andreas Zedrosser, Jon E. Swenson, F. Pelletier

Corresponding author: Martin Leclerc, Martin.Leclerc2@USherbrooke.ca
Département de Biologie, Université de Sherbrooke, Sherbrooke, Québec, Canada, J1K 2R1

Supplementary materials:

Appendix 1 Details of the monitoring protocol.

Appendix 2 Map of the distribution of monitored and hunter-killed bears in Sweden.

Appendix 3 Table of descriptive statistics of monitored and hunting records datasets.

Appendix 4 Predictions of hunter-killed and monitored yearling body mass from 1996 to 2013.

Appendix 5 Predictions of hunter-killed and monitored adult female body mass from 1996 to 2013.

Appendix 6 Supplementary analyses on age structure and mass of monitored and hunter-killed females.

Appendix 1

Details of the capture and monitoring protocols

Females without young and females accompanied by yearlings were immobilized with a dart gun from a helicopter. Females with cubs of the year were not captured for animal welfare reasons. Captures were carried out after den emergence from mid-April to early May. Because all bears were captured within a 2-week period, we did not adjust body size for capture date. All females were marked individually with tattoos (inside the upper lip), and passive integrated transponder (PIT) tags under anesthesia. Females were fitted with radio transmitters, radio-implants (Telonics, model IMP/40/L HC), or both. Females were originally fitted with VHF radio transmitters (Telonics, model 500). However, since 2003, most (gradually from 6% to 90%) females captured or recaptured were fitted with GPS–GMS transmitters (GPS Plus, Vectronic Aerospace GmbH). A vestigial premolar tooth was collected from all females not captured as a yearling to estimate age based on the cementum annuli in the root (Mattson's Inc., Milltown, MT). Approximately 50–80% of the females in the study area were monitored annually (Scandinavian Brown Bear Project, personal communication). For further information about capture and handling of bears, see Arnemo et al. (2011) and Zedrosser et al. (2007).

Arnemo JM, Evans A, Fahlman A. 2011 Biomedical protocols for free-ranging brown bears, wolves, wolverines and lynx. Trondheim, Norway: Directorate for Nature Management.

Zedrosser A, Støen O-G, Sæbø S, Swenson JE. 2007 Should I stay or should I go? Natal dispersal in the brown bear. Anim. Behav. 74, 369–376. (doi:10. 1016/j.anbehav.2006.09.015)

Appendix 2

Map of the distribution of monitored (black triangles) and kill locations of hunter-killed (red stars) brown bears in Sweden, 1996-2013. More than 95% of the area covered by monitored bears is within the area of hunter-killed bears. The MCP centroids are 43 km apart. Note that we only show the home range centroids of the monitored bears (black triangles), but the bears' actual home ranges often extended beyond the MCP of monitored bears in the figure below, and thus, the spatial overlap is underestimated.

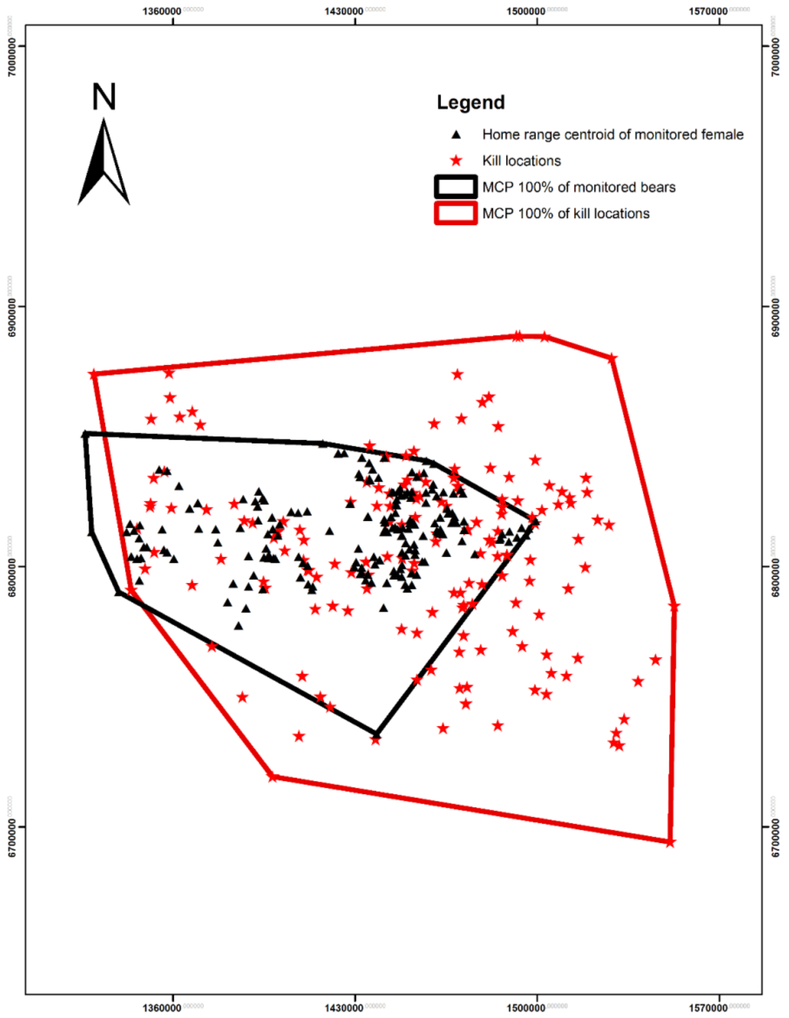

Appendix 3

Means of parameters used, 95% confidence intervals [lower:upper], and number of observations of brown bears in the monitored dataset and hunting records in Sweden, 1996-2013.

	Monitored dataset	Hunting records
Mean ratio of yearlings to adult females	1.32	0.71
Mean yearling mass (kg)	22 [10:37] (*n*=266)	54 [31:79] (*n*=108)
Mean adult female mass (kg)	84 [48:112] (*n*=205)	125 [78:177] (*n*=157)
Mean age of females	9 [4:20] (*n*=205)	8 [4:17] (*n*=157)

Appendix 4

Predictions for the mass of yearling male brown bears (solid line) and the 95% confidence intervals of the final model (transformed back to the original scale) suggesting bias in the data from hunter-killed brown bears in Sweden, 1996-2013. We investigated bias in yearling mass in the data from hunting records (red line and dots) in comparison to data from bears monitored by the Scandinavian Brown Bear Project (black line and dots).

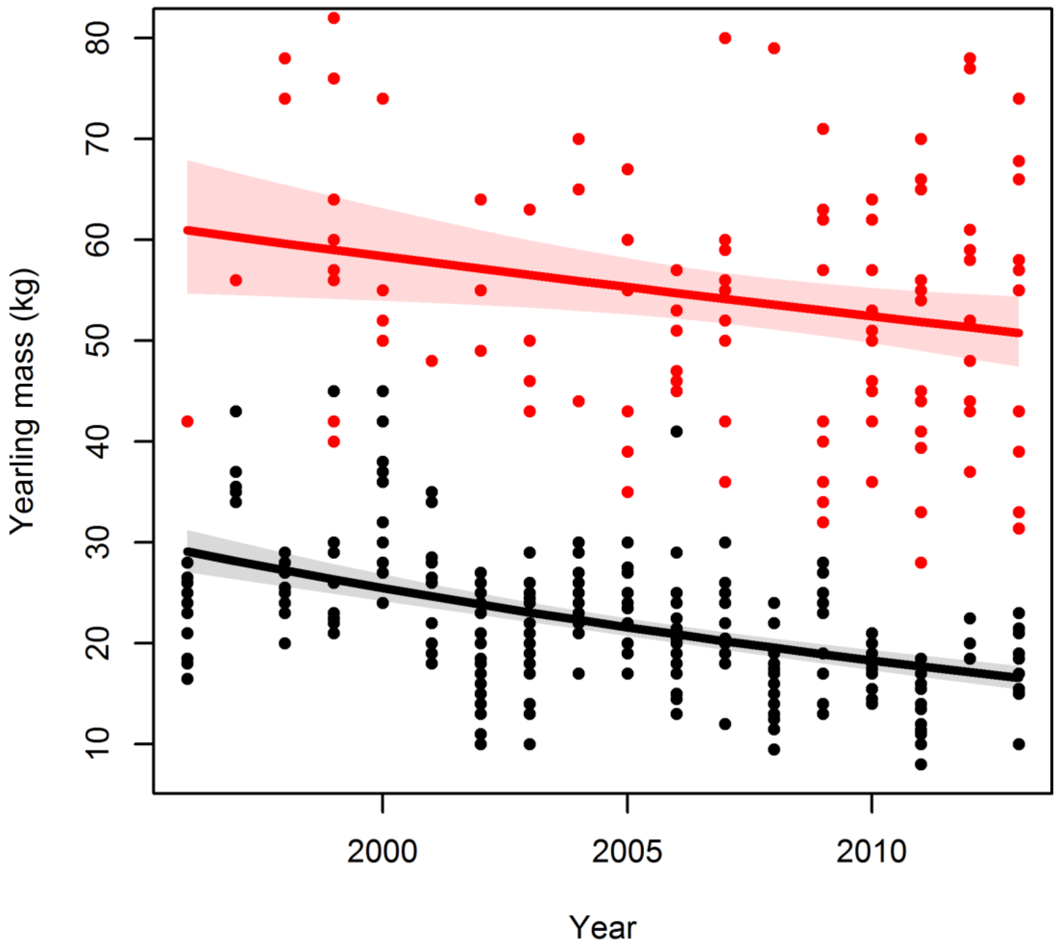

Appendix 5

Predictions (solid lines) for 8 year-old females and 95% confidence intervals of the final model (transformed back to the original scale) showing bias in data from hunter-killed brown bears in Sweden, 1996-2013. We investigated bias in adult female mass from the harvest records (red line and dots) in comparison to data from bears monitored by the Scandinavian Brown Bear Project (black line and dots).

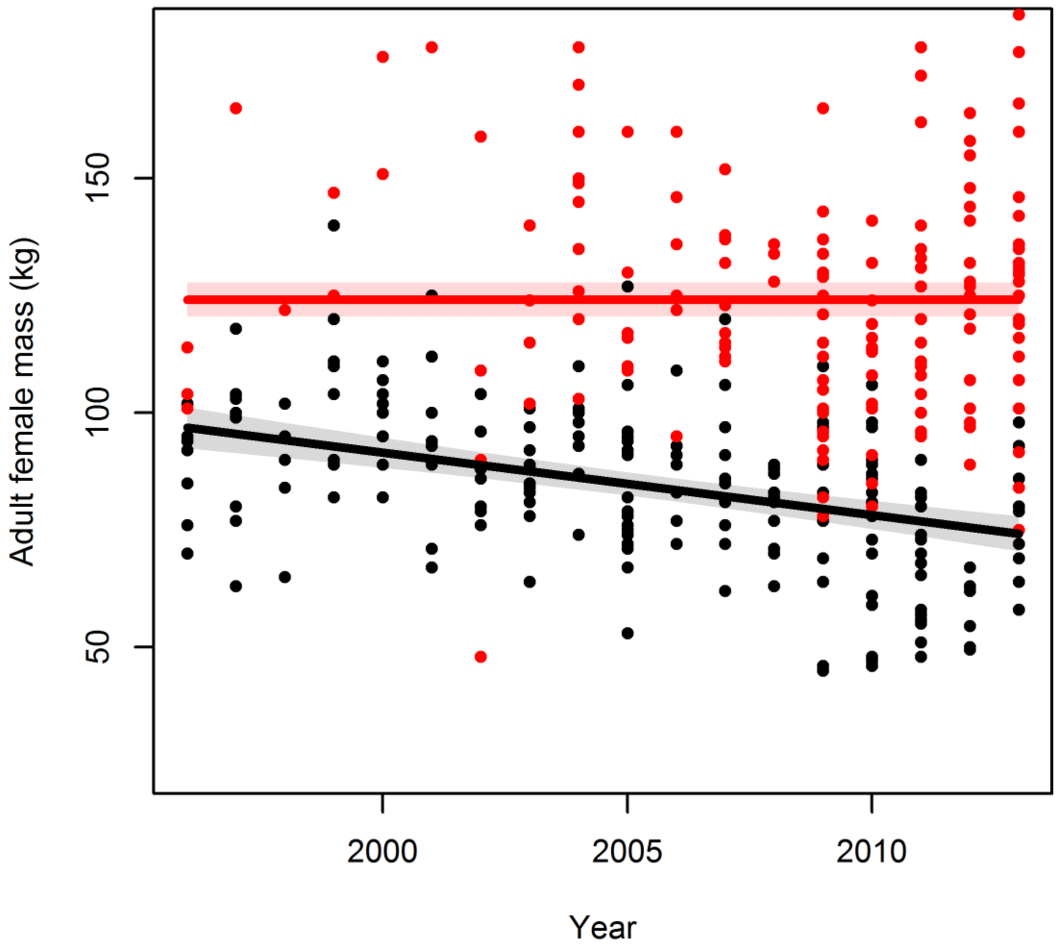

Appendix 6

Supplementary analyses showing that our results were not driven by 1) the fact that body mass was recorded during spring for monitored females and during autumn for hunter-killed females, or by 2) different age structures between monitored and hunter-killed datasets.

The difference in temporal trends between the monitored and hunter-killed female mass could potentially be affected by the fact that they were recorded at different times of the year. We therefore carried out a post-hoc analysis using only females that were monitored in spring and killed in autumn during the same year (n=20). We obtained a significant correlation of r = 0.87 (p < 0.001) between the mass in autumn (hunting dataset) and the mass in spring (monitoring dataset). The mass in spring explained 74.5% of the mass variation in autumn and this relationship was not driven by female age (p = 0.102 and VIF=1.56), suggesting that a small female in spring would also be small in autumn. Consequently, we argue that the different temporal trends observed between datasets were not caused by the fact that bears were weighed at different times of the year.

The observed difference in temporal trends in female mass between the monitored and hunter-killed datasets could potentially have been a result of a different age structure between the datasets. However, a post-hoc analysis showed that age class distribution was similar between the two datasets (X^2 = 21.839, df = 19, p = 0.29).

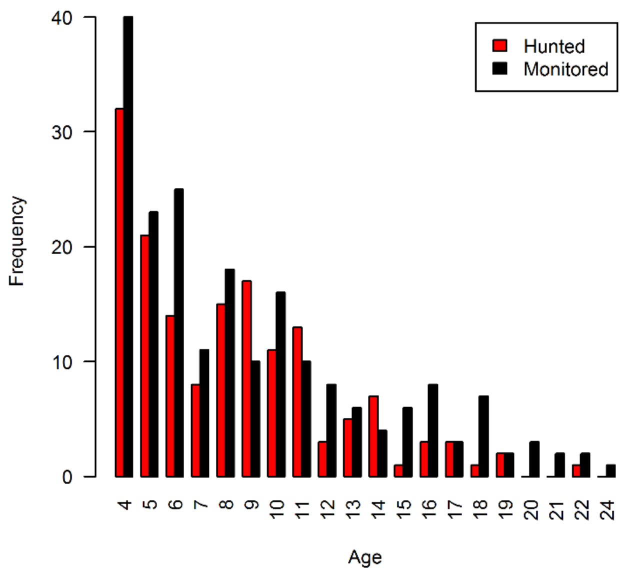

Supplement: Supplementary materials [file rsbl20160197supp1.pdf]
